# Large language models will not replace healthcare professionals: curbing popular fears and hype

**DOI:** 10.1177/01410768231173123

**Published:** 2023-05-18

**Authors:** Arun James Thirunavukarasu

**Affiliations:** Corpus Christi College, University of Cambridge, Cambridge, CB2 1RH, UK

Following the release of ChatGPT, large language models (LLMs) have entered the mainstream. ChatGPT and GPT-4 recently garnered particular attention for attaining expert-level performance in United States Medical Licensing Examinations.^1^ However, performance is not perfect, and has not been as impressive in more specialised tests, such as the Membership of the Royal College of General Practitioners Applied Knowledge Test.^1,2^ ChatGPT frequently ‘hallucinates’, providing false, unverified information in the same manner as which it delivers facts. While performance in clinical tasks is expected to improve dramatically with the release of GPT-4, remaining inaccuracy and lack of an uncertainty indicator preclude autonomous deployment of ChatGPT and LLM chatbots like it in clinical settings.^2,3^

LLM applications may nevertheless revolutionise cognitive work – tools such as ChatGPT excel in tasks where specialist knowledge is not required, or is provided by the user prompt: examples include correcting language and rephrasing information for different audiences or within other constraints (e.g. word limits), and it has already been proposed as a tool for administrative tasks, clinical work and patient education.^4^ While this does represent an impressive advance in natural language processing, and benefits may be manifold across fields including medicine, these limited use-cases do not live up to the hype surrounding LLMs and artificial intelligence (AI) more generally in 2023. This is due to a fundamental misunderstanding about the form of AI represented by LLMs.

Do LLMs represent artificial generalised intelligence (AGI)? The answer is currently *probably not*, despite emergence of interactive conversational interfaces and few-shot or zero-shot properties – where models execute tasks that they have previously been exposed to only a few times before, or never before, respectively.^5,6^ This is demonstrated by observing how these models are trained, and the composition of their architecture. The backend LLM (GPT-3, from which GPT-3.5 was developed) underpinning older versions of ChatGPT was initially trained on a dataset of billions of words taken from books, Wikipedia and the wider internet.^5^ Through a process of machine learning, the GPT-3 accurately encoded the association between individual words in the training dataset.^5^ Through ‘reinforcement learning from human feedback’, GPT-3 was subsequently finetuned to provide appropriate responses to users’ queries – producing GPT-3.5.^7^

Through these processes, ChatGPT has developed an impressive ability to respond appropriately to diverse prompts, albeit equally lucidly with accurate and inaccurate statements. This lucidity, responsiveness and flexibility have led to sensational claims regarding attainment of AGI that could feasibly replace professionals in cognitive roles.^6^ The performance of GPT-4 – which powers newer versions of ChatGPT – dwarfs that of GPT-3.5 across tasks including logical reasoning and medical aptitude tests.^1,3^ Moreover, GPT-4 can be prompted to adopt different roles on demand, and will accept multimodal input, processing images as well as text.^3^ Prominent figures in industry and academia have advocated for a moratorium on development of more advanced AI systems in response to concerns regarding safety, ethics and fears of replacement.^8^

Despite these fears and hype, the barriers to implementation of LLMs replacing healthcare professionals in any capacity still look out of reach. Although GPT-4’s architecture and training are confidential, it likely relies on similar schemata to its predecessor as it exhibits similar (albeit fewer) hallucinations and reasoning errors, including in medicine.^1–3,6^ None of ChatGPT’s published autonomous training involved actual comprehension of language in context; the meaning (as we understand it) of words in the dataset was immaterial throughout.^7^ While this brute force linguistic processing may prove sufficient to develop a form of AGI, it appears that these LLMs will continue to be afflicted by mistakes and errors.^6^ Fundamentally, LLMs are limited by the quality of information available for training or browsing in response to queries, which remains governed by human activity (e.g. research, policymaking). In addition, there is currently little reported development of integrating LLMs with physical agents that could replace the myriad of practical tasks undertaken in healthcare.

If future models can overcome their current shortcomings, they may prove to be more effective decision-makers than senior clinicians – this would ultimately have to be proven in large clinical trials pitting clinician decisions against AI decisions. However, even if sufficient technology is developed and controversial trials of clinical effectiveness can be conducted, clinicians will not be replaced. Patients distrust autonomous AI and benefit when human clinicians cater to their individual circumstances and values.^9,10^ Clinical practice requires more than merely responding accurately to queries or even optimising the biomedical process of diagnosis and management, and medical professionals are therefore not predicted to be overly exposed to changes induced by proliferation of LLM applications.^11^ Tools such as ChatGPT may be of great use in medicine, but AI is not ready to replace us!

## References

[1] Nori H King N McKinney SM Carignan D Horvitz E . Capabilities of GPT-4 on medical challenge problems. 2023. See http://arxiv.org/abs/2303.13375 (last checked 26 March 2023).

[2] Thirunavukarasu A Hassan R Mahmood S Sanghera R Barzangi K El Mukashfi M , et al. Trialling a large language model (ChatGPT) in general practice with the Applied Knowledge Test: observational study demonstrating opportunities and limitations in primary care. JMIR Med Educ 2023; 9: e46599. 37083633 10.2196/46599 PMC10163403

[3] OpenAI. GPT-4 Technical Report. 2023. doi:10.48550/arXiv.2303.08774

[4] Will ChatGPT transform healthcare? *Nat Med* 2023; 29: 505–506. 10.1038/s41591-023-02289-5 36918736

[5] Brown T Mann B Ryder N Subbiah M Kaplan J Dhariwal P , , et al. Language models are few-shot learners. In: Larochelle H, Ranzato M, Hadsell R, Balcan MF and Lin H, eds. Advances in Neural Information Processing Systems. Red Hook, NY: Curran Associates, Inc., 2020, pp.1877–1901.

[6] Bubeck S Chandrasekaran V Eldan R Gehrke J Horvitz E Kamar E , et al. Sparks of artificial general intelligence: early experiments with GPT-4. *arXiv* 2023. doi:10.48550/arXiv.2303.12712

[7] Ouyang L Wu J Jiang X Almeida D Wainwright CL Mishkin P , , et al. Training language models to follow instructions with human feedback. *arXiv* 2022.

[8] Pause Giant AI Experiments: an open letter. Future of Life Institute. 2023. See https://futureoflife.org/open-letter/pause-giant-ai-experiments/ (last checked 2 April 2023).

[9] Esmaeilzadeh P Mirzaei T Dharanikota S. Patients’ perceptions toward human–artificial intelligence interaction in health care: experimental study. J Med Internet Res 2021; 23: e25856. 34842535 10.2196/25856 PMC8663518

[10] Epstein RM Street RL. The values and value of patient-centered care. Ann Fam Med 2011; 9: 100–103. 21403134 10.1370/afm.1239 PMC3056855

[11] Eloundou T Manning S Mishkin P Rock D. GPTs are GPTs: an early look at the labor market impact potential of large language models. 2023. See http://arxiv.org/abs/2303.10130 (last checked 27 March 2023).

